# Comparison of the Diagnostic Value of Immunochromatography Kits in Corona Virus Disease 2019 Patients: A Prospective Pilot Study

**DOI:** 10.31662/jmaj.2020-0084

**Published:** 2021-01-14

**Authors:** Toshiya Mitsunaga, Yutaka Seki, Masakata Yoshioka, Ippei Suzuki, Kumi Akita, Syunsuke Mashiko, Masahiko Uzura, Satoshi Takeda, Akihiro Sekine, Kunihiro Mashiko

**Affiliations:** 1 Department of Emergency Medicine, Association of EISEIKAI Medical and Healthcare Corporation Minamitama Hospital, Tokyo, Japan; 2 Department of Emergency Medicine, Jikei University School of Medicine, Tokyo, Japan; 3 Center for Preventive Medical Sciences, Chiba University, Chiba, Japan; 4 Department of Cardiology, Association of EISEIKAI Medical and Healthcare Corporation Minamitama Hospital, Tokyo, Japan; 5 Department of Internal Medicine, Association of EISEIKAI Medical and Healthcare Corporation Minamitama Hospital, Tokyo, Japan; 6 Department of Emergency and Critical Care Medicine, Chiba University, Chiba, Japan

**Keywords:** COVID-19, antibody, IgM, IgG, immunochromatography kit

## Abstract

**Introduction:**

The spread of coronavirus 2019 (COVID-19) is a serious problem all over the world. Several immunochromatography kits of the antibody for severe acute respiratory syndrome coronavirus 2 (SARS-CoV-2) have been developed, but it is still unclear which kits have high diagnostic value. This study aims to evaluate the accuracy rate for antibody detection of each immunochromatography kit and reveal which kit has a high diagnostic value for antibody detection.

**Methods:**

This study was carried out between 1 August 2020 and 14 October 2020 at the Association of EISEIKAI Medical and Healthcare Corporation Minamitama Hospital. Patients diagnosed with COVID-19 and approximately 30 days after symptom onset were included as the positive group. The serum SARS-CoV-2 antibodies were analysed using seven immunochromatography kits.

**Results:**

Twenty samples (Positive group: 10 patients, Negative group: 10 healthy medical workers) were included in this study. The median age of the patients was 44 years, and the median duration from symptom onset was 30.5 days in the positive group. The accuracy rates for IgM/IgG detection were: 90.0%/100% in Kit A; 50.0%/95.0% in Kit B; 55.0%/65.0% in Kit C; 60.0%/55.0% in Kit D; 50.0%/80.0% in Kit E; 80.0%/90.0% in Kit F; and 90.0%/100% in Kit G.

**Conclusions:**

Our study showed that there is a variation of accuracy rates between immunochromatography kits for antibodies of SARS-CoV-2. COVID-19 IgG/IgM RAPID TEST CASSETTE (Hangzhou Biotest Biotech Co., Ltd., China) and Nadal COVID-19 IgG/IgM Rapid Test (BioServUK Ltd., UK: United Kingdom) have high accuracy rates for both IgM and IgG detection. Evidence from large population studies of immunochromatography kits is needed to clarify the details of diagnostic value for SARS-CoV-2.

## Introduction

In December 2019, the first group of patients with unknown-caused pneumonia was confirmed in Wuhan, China. The pathogen was detected as severe acute respiratory syndrome coronavirus 2 (SARS-CoV-2). The spread of coronavirus 2019 (COVID-19) was so fast that it has been very difficult to control the further infection of COVID-19 since the World Health Organization declared a pandemic. In Japan, The Ministry of Health, Labor and Welfare of Japan announced 71,856 cases of COVID-19, with 1,363 deaths (mortality rate: 1.89%) confirmed by 7 September 2020 ^(1)^.

Studies of severe acute respiratory syndrome (SARS) and Middle East respiratory syndrome carried out by Hsueh et al. and Corman et al. revealed that virus antibodies were detected in more than 80% of patients at two weeks after symptom onset ^(2), (3)^.

Similarly, some reports have shown that SARS-CoV-2 immunoglobulin-M (IgM) and immunoglobulin-G (IgG) positive detection rates gradually increased after infection and reached more than 80% at 15 days after symptom onset, whereas the IgM positive detection rate decreased after 20 days ^(4), (5)^. On the other hand, a study carried out by Long et al. showed that both the IgM and IgG positive detection rates gradually increased and reached more than 90% in IgM and 100% in IgG at 20 days after symptom onset without a decline of positive detection rates ^(6)^.

While several immunochromatography kits for the antibody of SARS-CoV-2 have been developed, it is still unclear which immunochromatography kit has a high accuracy rate for anti-SARS-CoV-2 antibody detection, with there being a variation in antibody-positive detection rates for SARS-CoV-2 between antibody tests. 

The aim of this study is to evaluate the accuracy rate for antibody detection of each immunochromatography kit and reveal which kit has a high diagnostic value for antibody detection.

## Materials and Methods

### Study design

This prospective pilot single-centre observational study was carried out to evaluate the diagnostic value of immunochromatography kits in COVID-19 patients. The protocol for this research project was approved by a suitably constituted Ethics Committee of the institution and conforms to the provision of the Declaration of Helsinki (Committee of Association of the EISEIKAI Medical and Healthcare Corporation Minamitama Hospital, Approval No. 2020-Ack-05), and written consent was obtained from all the patients.

### Study setting and population

The present study was carried out between 14 August 2020 and 14 October 2020 at the Association of EISEIKAI Medical and Healthcare Corporation Minamitama Hospital, a secondary emergency medical institution. Patients diagnosed as COVID-19 by real-time polymerase chain reaction (RT-PCR) and about 30 days after symptom onset were included in this study as the positive group. Healthy medical workers who did not have an episode of infection for the past six months and whose RT-PCR was negative were included in this study as the negative group. Information on age, sex, duration from onset, symptoms, body mass index, comorbidity, pneumonia, antiviral drugs, inhaled oxygen, and hospitalisation were recorded.

### Immunochromatography kits

The serum SARS-CoV-2 antibodies of the patients were analysed using seven immunochromatography kits. (1) Kit A: COVID-19 IgG/IgM RAPID TEST CASSETTE (Hangzhou Biotest Biotech Co., Ltd., China); (2) Kit B: 2019-nCoV IgG/IgM Test Card (Lumiquick Diagnostics Inc., US); (3) Kit C: Coronavirus (COVID-19) IgM/IgG Rapid Test Kit (RayBiotech Life Inc., US); (4) Kit D: COVID-19 Human IgM/IgG Rapid Test (Abnova Co., Ltd., Taiwan); (5) Kit E: GenBody COVID-19 IgM/IgG (GenBody Inc., Korea); (6) Kit F: STANDARD STANDARDTM Q COVID-19 IgM/IgG Duo Test (SD BIOSENSOR Inc., Korea); and (7) Kit G: Nadal COVID-19 IgG/IgM Rapid Test (BioServUK Ltd., UK: United Kingdom). Product information of the seven immunochromatography kits is summarised in Table 1. Two kits targeted spike protein, three kits targeted nucleocapsid protein, and two kits targeted spike and nucleocapsid protein. Whole blood, serum, or plasma samples can be used as specimens in all kits. Five kits require 10-20 μL of sample volume, but the other kits require only 2 μL or 2-3 drops of a mixture of the sample (25 μL) and diluent. All seven kits detect anti-SARS-CoV-2 IgM and IgG antibodies separately within 10-20 minutes. Cross-reactivity with antibodies to other coronaviruses (HKU1, NL63, OC43, 229E) was confirmed in five kits, and cross-reactivity to Rheumatoid factor was confirmed in two kits. More than half of the kits were not affected by blood compounds or common drugs. According to the recommendation of the manufacturer’s instructions, weak positive reactions of kits were considered as positive.

**Table 1.** Product Description of the Seven Immunochromatography Kits of COVID-19.

	1) COVID-19 IgG/IgM RAPID TEST CASSETTE (Kit A)	2) 2019-nCoV IgG/IgM Test Card (Kit B)	3) Coronavirus (COVID-19) IgM/IgG Rapid Test Kit (Kit C)	4) COVID-19 Human IgM/IgG Rapid Test (Kit D)	5) GenBody COVID-19 IgM/IgG (Kit E)	6) STANDARD STANDARDTM Q COVID-19 IgM/IgG Duo Test (Kit F)	7) Nadal COVID-19 IgG/IgM Rapid Test (Kit G)
Targeting antibody	IgM and IgG	IgM and IgG	IgM and IgG	IgM and IgG	IgM and IgG	IgM and IgG	IgM and IgG
Qualitative analysis	Yes	Yes	Yes	Yes	Yes	Yes	Yes
Protein labeled	Spike protein	Spike protein S1 domain Nucleocapsid protein	Nucleocapsid protein	Nucleocapsid protein Receptor binding domain (RBD) protein	Nucleocapsid protein	Nucleocapsid protein	Spike protein
Specimen type(s)	Whole blood, serum or plasma	Whole blood, serum or plasma	Whole blood, serum or plasma	Whole blood, serum or plasma	Whole blood, serum or plasma	Whole blood, serum or plasma	Whole blood, serum or plasma
Specimen amount required	10 μL	2 μL	Add 25 μL of sample to the Diluent, and add 2-3 drops to the pad section.	Whole blood: 20 μL Serum or plasma: 10 μL	Whole blood: 20 μL Serum or plasma: 10 μL	Whole blood: 20 μL Serum or plasma: 10 μL	10 μL
Turnaround time	10 min	15 min	20 min	15 min	10 min	10-15 min	10 min
Sensitivity (95% CI)	IgM: 91.8% (83.8-96.6%) IgG: 100.0% (96.1-100.0%)	87.8%	84.1%	91.89%	91.7%	94.51%	94.1% (86.8-98.1%)
Specificity (95% CI)	IgM: 99.2% (97.7-99.8%) IgG: 99.5% (98.1-99.9%)	99.0%	92.3%	100.00%	97.5%	95.74%	99.2% (97.7-99.8%)
Accuracy (95% CI)	IgM: 97.8% (96.0-98.9%) IgG: 99.6% (98.4-99.9%)	96.8%	NA	NA	96.5%	NA	98.2% (96.6-99.2%)
Limit of detection	NA	NA	NA	NA	NA	IgM: 9.37 μg/mL IgG: 3.75 μg/mL	NA
Confirmed no cross reactivity with antibodies to non-coronaviruses	Influenza A and B, RSV, Adenovirus, HBV, Syphilis, H. Pylori, HIV, HCV, HANA	Influenza A and B, Adenovirus, Rotavirus, Mycoplasma Pneumoniae	Influenza A and B, RSV, HBV, HCV	Influenza A and B, RSV	RSV IgG, Mycoplasma pneumonia IgM/IgG, HCV, HIV, Dengue, Zika IgG, Chikungunya IgG, Yellow fever IgG, Adenovirus IgM, Leptospira IgG	HIV, Japanese Encephalitis, Zika virus, Chikungunya, Dengue, Salmonella typhi IgM, Rubella IgM, CMV IgM/IgG, Tick borne encephalitis IgM, West Nile virus, Treponema palladium, HAV IgM/IgG, HBV Ab, HCV Ab, Influenza vaccine, Leishmania, Brucella IgM, Chagas, Toxoplasma, Filariasis, Mycoplasma pneumonia IgM/IgG, Influenza A IgM, Influenza B IgM, Influenza A and B IgG+IgM, Tuberculosis	Influenza A and B, RSV, Adenovirus, HBV, T. pallidum, H. pylori, HIV, HCV, HANA
Cross reactivity with antibodies to other coronaviruses	HKU1, NL63, OC43, 229E	NA	HKU1, NL63, OC43, 229E	HKU1, NL63, OC43, 229E	HKU1, NL63, OC43, 229E	HKU1, NL63, OC43, 229E	HKU1, NL63, OC43, 229E
Cross reactivity with antibodies to SARS or MERS	MERS-CoV	NA	NA	NA	NA	NA	SARS-CoV, MERS-CoV
Confirmed no cross reactivity with antibodies to blood compounds	Hemoglobin: 1000 mg/dL Albumin: 2 g/dL Bilirubin: 1 g/dL Uric acid: 20 mg/mL Creatine: 200 mg/dL	Rheumatoid Factor: 80 IU/mL Hemoglobin: 10 mg/mL Bilirubin: 342 μmol/L Triglyceride: 37 mmol/L	NA	NA	NA	Hemoglobin, Triglycerides, Cholesterol, Bilirubin	Hemoglobin: 10000 mg/dL Albumin: 20 g/dL Bilirubin: 10000 mg/dL Uric acid: 20 mg/mL Creatine: 2000 mg/L
Cross reactivity with antibodies to blood compounds	Rheumatoid Factor	NA	NA	NA	NA	NA	Rheumatoid Factor
Confirmed no cross reactivity with antibodies to common drugs	Acetaminophen: 20 mg/dL Acetylsalicylic Acid: 20 mg/dL Ascorbic Acid: 2g/dL Caffeine: 20 mg/dL Ethanol: 1% Gentistic acid: 20 mg/dL Oxalic acid: 60 mg/dL	Histamine Hydrochloride, Interferon-α, Zanamivir, Ribavirin, Oseltamivir, Peramivir, Lopinavir, Ritonavir, Arbidol, Levofloxacin, Azithromycin, Ceftriaxone, Meropenem, Tobramycin	NA	NA	NA	Zanamivir, Oseltamivir, Artemether-lumefantrine, Doxycycline hyclate, Quinine, Lamivudine, Ribavirin, Daclatasvir, Acetaminophen, Acetylsalicylic acid, Ibuprofen, Erythromycin, Ciprofloxacin, Caffeine, Ethanol, Biotin	Acetaminophen: 200 mg/L Acetylsalicylic Acid: 200 mg/L Ascorbic Acid: 20000 mg/L Caffeine: 200 mg/L Ethanol: 1% Gentistic acid: 200 mg/L Oxalic acid: 600 mg/L

RSV, respiratory syncytial virus; HBV, hepatitis B virus; HCV, hepatitis C virus; HIV, human immunodeficiency virus; CMV, cytomegalovirus; SARS, severe acute respiratory syndrome; MERS, Middle East respiratory syndrome; NA, not available

### Statistical analyses

Continuous variables were described as medians and interquartile ranges (IQR) and were compared with Mann-Whitney *U*-test. Categorical variables were described as numbers and percentages and were compared with Fisher’s exact test. A *p*-value of less than 0.05 was considered to indicate statistical significance. Data were analysed in the Statistical Package for the Social Sciences, version 26.0 (SPSS, Chicago, IL, USA).

## Results

Twenty samples (Positive group: 10 patients, Negative group: 10 healthy medical workers) were included in this study. The median age (interquartile range) of the samples was 44.0 (25.3) years in the positive group and 39.5 (7.0) years in the negative group. Six (60%) samples in the positive group and in the negative group were male. The major chief complaints were fever (8 cases: 80%), fatigue (7 cases: 70%), and cough (6 cases: 60%). The median duration from symptom onset was 30.5 (IQR = 4) days. Four patients (40%) had some comorbidities as follows: hypertension (2 cases: 20%); hyperlipidaemia (1 case: 10%); diabetes mellitus (2 cases: 20%); COPD (chronic obstructive pulmonary disease)/asthma (1 case: 10%); CKD (chronic kidney disease) (1 case: 10%); and liver disease (1 case: 10%). On the other hand, no one had comorbidity in the negative group. Seven patients (70%) were hospitalised in our hospital, one patient (10%) was hospitalised in another hospital, four pneumonia cases (40%) were detected by radiological tests, and one case (10%) was supplied oxygen. One case (10%) was administered Favipiravir, and two cases (20%) were administered Remdesivir (Table 2). In the positive group, the IgM positive detection rates were eight cases (80%) in Kit A, zero (0) cases (0%) in Kit B, one case (10%) in Kit C, two cases (20%) in Kit D, zero (0) cases (0%) in Kit E, six cases (60%) in Kit F, and eight cases (80%) in Kit G. 

**Table 2.** Baseline Characteristics of The Study Population.

	Positive Group Median (interquartile range) (n=10)	Negative Group Median (interquartile range) (n=10)	p-value
Age, years	44.0 (25.3)	39.5 (7.0)	0.37
Sex [n (%)]		
Male	6 (60)	6 (60)	1.00
Female	4 (40)	4 (40)
Duration from onset, days	30.5 (4.0)	-	-
Symptoms [n (%)]			
Fever	8 (80)	-	-
Cough	6 (60)	-	-
Fatigue	7 (70)	-	-
Sore throat	4 (40)	-	-
Nausea/Vomit	2 (20)	-	-
Diarrhea	4 (40)	-	-
Headache	4 (40)	-	-
Loss of appetite	4 (40)	-	-
Body aches	3 (30)	-	-
Olfactory disorder	5 (50)	-	-
Taste disorder	4 (40)	-	-
BMI (Body mass index), kg/m2	23.5 (8.9)	20.9 (2.4)	0.09
Comorbidity [n (%)]			
Hypertension	2 (20)	-	-
Hyperlipidaemia	1 (10)	-	-
Diabetes mellitus	2 (20)	-	-
COPD/Asthma	1 (10)	-	-
CKD	1 (10)	-	-
Liver diseases	1 (10)	-	-
Pneumonia [n (%)]	4 (40)	-	-
Antiviral drugs [n (%)]			
Favipiravir	1 (10)	-	-
Remdesivir	2 (20)	-	-
Inhaled Oxygen [n (%)]	1 (10)	-	-
Hospitalisation [n (%)]	8 (80)	-	-

COPD, Chronic obstructive pulmonary disease; CKD, Chronic kidney disease

**Figure 1.**
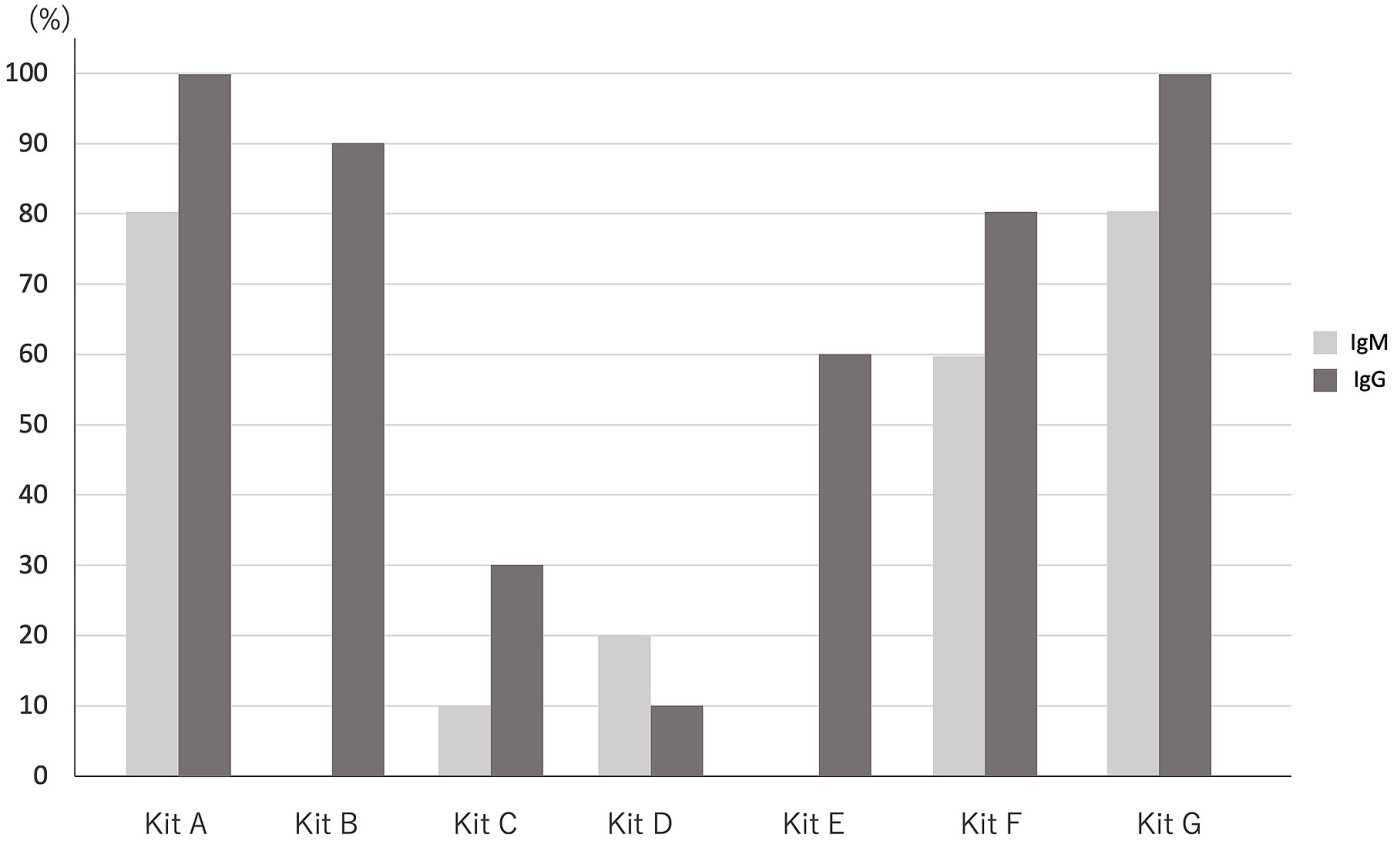
The positive detection rates of IgM and IgG for SARS-CoV-2.

Moreover, the IgG positive detection rates were ten cases (100%) in Kit A, nine cases (90%) in Kit B, three cases (30%) in Kit C, one case (10%) in Kit D, six cases (60%) in Kit E, eight cases (80%) in Kit F, and ten cases (100%) in Kit G in the same group (Figure 1). On the contrary, there was no positive reaction of IgM and IgG in the negative group. Further detailed clinical information of each patient in the positive group is shown in Table 3, and the details of the reaction for antibodies of SARS-CoV-2 in the positive group are shown in Table 4. The number of kits that have antibodies positive reaction is significantly larger in patients older than 40 years than in patients younger than 40 years (median [IQR]: 3 [0.5] vs. 5.5 [1], *p* < 0.05). The number of kits that have antibodies positive reaction was larger in the patients who had pneumonia, were administered antiviral drugs (Favipiravir or Remdesivir), or were supplied oxygen, but there was no significant difference (median [IQR]: 4 [2] vs. 6 [1], *p* = 0.054).

**Table 3.** The Detailed Clinical Information of the Ten Patients in the Positive Group.

Patients No. gender (age)	Duration post symptom onset (Days)	BMI(kg/m2)	Comorbidity						Pneumonia	Antiviral drugs		Inhaled O2	Hospitalisation
			HT	HL	DM	COPD/asthma	CKD	Liver diseases		Favipiravir	Remdesivir		
Patients 1 male (27)	43	19.8	-	-	-	-	-	-	-	-	-	-	+
Patients 2 female (30)	35	20.2	-	-	-	-	-	-	-	-	-	-	-
Patients 3 male (56)	32	23.1	-	-	-	-	-	+	-	-	-	-	-
Patients 4 male (60)	33	26.5	-	-	-	+	-	-	+	-	+	-	+
Patients 5 female (30)	29	17.5	-	-	-	-	-	-	-	-	-	-	+
Patients 6 male (45)	29	35.8	+	-	+	-	+	-	-	+	-	-	+
Patients 7 male (64)	29	32.9	+	-	+	-	-	-	+	-	-	-	+
Patients 8 female (53)	29	23.8	-	-	-	-	-	-	-	-	-	-	+
Patients 9 female (25)	33	16.8	-	-	-	-	-	-	+	-	-	-	+
Patients 10 male (43)	29	29.6	-	+	-	-	-	-	+	-	+	+	+

BMI, Body Mass Index; HT, Hypertension; HL, Hyperlipidaemia; DM, Diabetes mellitus; COPD, Chronic obstructive pulmonary disease; CKD, Chronic kidney disease

**Table 4.** THe Details of the Reaction in Seven Immunochromatography Kits for Antibodies of SARS-CoV-2.

Patients No.gender (age)	1) COVID-19 IgG/IgM RAPID TEST CASSETTE (Kit A)		2) 2019-nCoV IgG/IgM Test Card (Kit B)		3) Coronavirus (COVID-19) IgM/IgG Rapid Test Kit (Kit C)		4) COVID-19 Human IgM/IgG Rapid Test (Kit D)		5) GenBody COVID-19 IgM/IgG (Kit E)		6) STANDARD STANDARDTM Q COVID-19 IgM/IgG Duo Test (Kit F)		7) Nadal COVID-19 IgG/IgM Rapid Test (Kit G)	
	IgM	IgG	IgM	IgG	IgM	IgG	IgM	IgG	IgM	IgG	IgM	IgG	IgM	IgG
Patients 1 male (27)	+	+	-	+	-	-	-	-	-	-	-	-	+	+
Patients 2 female (30)	-	+	-	+	-	-	-	-	-	-	-	-	-	+
Patients 3 male (56)	++	++	-	+	-	-	-	-	-	-	+	+	++	++
Patients 4 male (60)	++	++	-	+	+	+	-	-	-	+	+	+	++	++
Patients 5 female (30)	+	++	-	+	-	-	-	-	-	+	+	+	+	++
Patients 6 male (45)	++	++	-	+	-	+	+	+	-	+	+	++	++	++
Patients 7 male (64)	++	++	-	+	-	+	-	-	-	+	+	++	++	++
Patients 8 female (53)	+	++	-	+	-	-	+	-	-	+	-	++	+	++
Patients 9 female (25)	-	+	-	-	-	-	-	-	-	-	-	+	-	+
Patients 10 male (43)	++	++	-	+	-	-	-	-	-	+	+	+	++	++

(-), no reaction; (+), weak reaction; (++), strong reaction

The sensitivity, specificity, false positive rate, false negative rate, and accuracy rate are shown in Table 5. The accuracy rates of Kit A and Kit G were high: 90.0% for IgM detection; and 100% for IgG detection, respectively. On the contrary, the accuracy rates of Kit B, Kit C, Kit D, and Kit E for IgM detection were low (50%-60%), and those of Kit C and Kit D for IgG detection were also low (55%-65%).

**Table 5.** The Quality in Seven Immunochromatography Kits for Antibodies of SARS-CoV-2

	1) COVID-19 IgG/IgM RAPID TEST CASSETTE (Kit A)	2) 2019-nCoV IgG/IgM Test Card (Kit B)	3) Coronavirus (COVID-19) IgM/IgG Rapid Test Kit (Kit C)	4) COVID-19 Human IgM/IgG Rapid Test (Kit D)	5) GenBody COVID-19 IgM/IgG (Kit E)	6) STANDARD STANDARDTM Q COVID-19 IgM/IgG Duo Test (Kit F)	7) Nadal COVID-19 IgG/IgM Rapid Test (Kit G)
IgM							
Sensitivity	80.0%	0%	10.0%	20.0%	0%	60.0%	80.0%
Specificity	100%	100%	100%	100%	100%	100%	100%
FPR	0%	0%	0%	0%	0%	0%	0%
FNR	20.0%	100%	90.0%	80.0%	100%	40.0%	20.0%
Accuracy	90.0%	50.0%	55.0%	60.0%	50.0%	80.0%	90.0%
IgG							
Sensitivity	100%	90.0%	30.0%	10.0%	60.0%	80.0%	100%
Specificity	100%	100%	100%	100%	100%	100%	100%
FPR	0%	0%	0%	0%	0%	0%	0%
FNR	0%	10%	70.0%	90.0%	40.0%	20.0%	0%
Accuracy	100%	95.0%	65.0%	55.0%	80.0%	90.0%	100%

FPR, False Positive Rate; FNR, False Negative Rate

## Discussion

In our study, Kit A) COVID-19 IgG/IgM RAPID TEST CASSETTE (Hangzhou Biotest Biotech Co., Ltd., China) and Kit G) Nadal COVID-19 IgG/IgM Rapid Test (BioServUK Ltd., UK: United Kingdom) have a high accuracy rate for detection of anti-SARS-CoV-2 IgM and IgG, and the accuracy rates were 90.0% for IgM detection and 100% for IgG detection. On the other hand, the accuracy rate for anti-SARS-CoV-2 antibodies detection was low in Kit C) Coronavirus (COVID-19) IgM/IgG Rapid Test Kit (RayBiotech Life Inc., US) and Kit D) COVID-19 Human IgM/IgG Rapid Test (Abnova Co., Ltd., Taiwan) (55.0%〜65.0%). These results suggest that there is a variation of accuracy rates for anti-SARS-CoV-2 antibodies detection between each kit, and it is desirable to use Kit A or Kit G for diagnosis of prior COVID-19 infection. To the best of our knowledge, this is the first analysis to compare the diagnostic value in more than five immunochromatography kits for anti-SARS-CoV-2 antibodies in Japan. 

In this study result showing that the accuracy rate for each kit was determined by false negative rate, the specificity of all the kits was 100%, and the sensitivity varies greatly between each kit. 

Two parts of protein are well known as the target of antibodies: spike (S) protein and nucleocapsid (N) protein. The S protein comprises S1 domain protein, S2 domain protein, and RNA binding domains protein ^(7)^. The N protein is composed of the N terminal domain and the C terminal domain ^(8)^. With antibodies to the N protein commonly being more sensitive than the S protein antibody for detecting SARS-CoV-2 infection ^(9)^, all the manufacturers provide information on the target of the antibody, but most of them are only written as the S protein or the N protein, and they do not show the detail of the target protein. Contrary to this result, our study showed that the accuracy of the kits targeting the S protein was higher than those targeting the N protein. There might be two reasons for this fact: 1) the accuracy rate of the kits is not related to the target proteins; or 2) the differences of the target proteins that are not shown on the product description are related to the reaction of antibodies.

A study carried out by Forster et al. showed that there are several genotypes in SARS-CoV-2 ^(10)^. As SARS-CoV-2 spreads in the world, not only genotypes but also S or N proteins mutate ^(11), (12)^. The manufacturers developed their rapid antibody kits based on the COVID-19 patients in their own countries, so the mutation of S protein or N protein might be the cause of false negative for the detection of antibodies.

The amount of the specimen has been set at 10 or 20 μL in most of the kits. On the other hand, the amount of the specimen is small in Kit B and Kit C. It is considered that the amount of specimen may not be directly related to the antibody detection, as, in this study, the IgG detection rate is as high as 80% despite the small amount of specimen in Kit B, whereas the IgG detection rate is as low as 20% despite the normal amount of specimen in Kit D. 

The limit of detection has been set in all the kits. However, we obtained the information about the limit of detection in one kit, so we could not compare between several kits. The limit of detection might be set low in the kits that have a high false negative rate. In our study, the number of kits that have antibody-positive reaction was significantly larger in patients older than 40 years (median [IQR]: 3 [0.5] vs. 5.5 [1], *p* < 0.05), with a study carried out by Jacofsky et al. showing that antibody was less likely to be produced after COVID-19 infection in a younger population ^(13)^. These results suggest that the concentration of antibodies of SARS-CoV-2 is higher in older patients.

In the study carried out by Chen et al*.*, antibody levels were correlated with the severity of COVID-19 pneumonia ^(14)^. On the other hand, the study carried out by Gozalbo-Rovira et al*.* showed that there was no association between antibodies of SARS-CoV-2 and COVID-19 severity ^(15)^.

In our study, the number of kits that have antibodies positive reaction was larger in the patients who had pneumonia, were administered antiviral drugs (Favipiravir or Remdesivir), or were supplied oxygen, but there was no significant difference (median [IQR]: 4 [2] vs. 6 [1], *p* = 0.054). 

These results may show that the concentration of antibodies of SARS-CoV-2 is higher in more severe cases.

Based on the above results, it could be said that the cause of the differences in diagnostic ability between each kit was mainly the limit values of the kits and the concentration of antibodies.

A study carried out by Jin et al. showed that the positive detection rate of antibody achieved the peak at 26-30 days after symptom onset ^(5)^, so we chose patients diagnosed as COVID-19 by RT-PCR and about 30 days after symptom onset as the positive group. Even if the RT-PCR is negative, the possibility of infection with COVID-19 remains. So, as the negative group, we chose healthy medical workers who did not have an episode of infection for the past six months and whose RT-PCR was negative.

Our study has several limitations. Firstly, we analysed the patient’s serum antibodies of SARS-CoV-2 only at one point, so the change of antibodies in a different phase is unclear. Secondly, the sample size was small, so there could be bias such as age or gender. Thirdly, we did not include the severe cases, and there could be severity bias. Further large-scale studies that evaluate antibodies of SARS-CoV-2 in several phases are needed in the future. 

In conclusion, our study showed that there is a variation of accuracy rates between immunochromatography kits for antibodies of SARS-CoV-2. COVID-19 IgG/IgM RAPID TEST CASSETTE (Hangzhou Biotest Biotech Co., Ltd., China) and Nadal COVID-19 IgG/IgM Rapid Test (BioServUK Ltd., UK: United Kingdom) had high accuracy rate for both IgM and IgG. Evidence from large population studies of immunochromatography kits is needed to clarify the details of the diagnostic value for COVID-19.

## Article Information

### Conflicts of Interest

None

### Acknowledgement

We are grateful for technical support from the Genesis Healthcare Co.

### Author Contributions

T.M., S.T., A.S., and K.M. designed the study. T.M., M.Y., K.A., I.S. and S.M. collected the data. T.M. and S.Y. performed the statistical analysis. T.M. and M.U. contributed to the final version of the manuscript. All authors discussed the results and contributed to the final manuscript.

### Approval by Institutional Review Board (IRB)

Approval No. 2020-Ack-05/Committee of Association of EISEIKAI Medical and Healthcare Corporation Minamitama Hospital.
